# The Role of *PALB2* in the DNA Damage Response and Cancer Predisposition

**DOI:** 10.3390/ijms18091886

**Published:** 2017-08-31

**Authors:** Thales C. Nepomuceno, Giuliana De Gregoriis, Francisco M. Bastos de Oliveira, Guilherme Suarez-Kurtz, Alvaro N. Monteiro, Marcelo A. Carvalho

**Affiliations:** 1 Programa de Pesquisa Clínica, Instituto Nacional de Câncer, Rio de Janeiro 20231-050, Brazil; thales.cn@gmail.com (T.C.N.); gregoriis@gmail.com (G.D.G.); kurtz@inca.gov.br (G.S.-K.); 2 Instituto de Biofísica Carlos Chagas Filho–Universidade Federal do Rio de Janeiro, Rio de Janeiro 21941-599, Brazil; francisco@biof.ufrj.br; 3 Cancer Epidemiology Program, H. Lee Moffitt Cancer Center & Research Institute, Tampa, FL 33612, USA; alvaro.monteiro@moffitt.org; 4 Instituto Federal do Rio de Janeiro-IFRJ, Rio de Janeiro 20270-021, Brazil

**Keywords:** Breast Cancer 1 (BRCA1), Breast Cancer 2 (BRCA2), Partner and Localizer of BRCA2 (PALB2), Fanconi Anemia Group N protein (FANCN), cancer predisposition, deoxyribonucleic acid (DNA) damage response, DNA double strand break, Fanconi anemia, homologous recombination

## Abstract

The deoxyribonucleic acid (DNA) damage response (DDR) is a major feature in the maintenance of genome integrity and in the suppression of tumorigenesis. PALB2 (Partner and Localizer of Breast Cancer 2 (BRCA2)) plays an important role in maintaining genome integrity through its role in the Fanconi anemia (FA) and homologous recombination (HR) DNA repair pathways. Since its identification as a BRCA2 interacting partner, PALB2 has emerged as a pivotal tumor suppressor protein associated to hereditary cancer susceptibility to breast and pancreatic cancers. In this review, we discuss how other DDR proteins (such as the kinases Ataxia Telangiectasia Mutated (ATM) and ATM- and Rad3-Related (ATR), mediators BRCA1 (Breast Cancer 1)/BRCA2 and effectors RAD51/DNA Polymerase η (Polη) interact with PALB2 to orchestrate DNA repair. We also examine the involvement of PALB2 mutations in the predisposition to cancer and the role of PALB2 in stimulating error-free DNA repair through the FA/HR pathway.

## 1. Introduction

During the late 1980s and early 1990s, investigators were on a pursuit for identifying the gene responsible for the predisposition to breast cancer. Hall and colleagues (1990) linked the early onset breast cancer to the long arm of chromosome 17 [1]. Four years later, Miki and colleagues (1994) mapped *BRCA1* (Breast Cancer 1, early onset), the first breast and ovarian cancer predisposing gene [1,2]. However, it became evident that *BRCA1* alone could not explain all cases of hereditary cancer. Soon after, a second predisposing breast cancer (BC) gene was positioned at chromosome 13q12–13, *BRCA2* [3,4]. It was already clear that both proteins played resembling biological functions, despite their lack of structural homology. By then, it was known that transgenic mice with a null genotype for *BRCA1* or *BRCA2* presented embryonic lethality. Data also indicated that both proteins were related to DNA damage repair through their interaction with RAD51 [5,6,7,8]. In 1998, Chen and colleagues demonstrated that BRCA1 and BRCA2 coexisted in the same protein complex but it was not clear whether this interaction was due to a direct or indirect association [9]. Despite significant efforts, a third predisposition gene has been sought for a long time without success.

Xia and colleagues (2006) described a new interaction partner for BRCA2, PALB2 (Partner and Localizer of BRCA2), responsible for BRCA2 nuclear localization and DNA damage response (DDR) functions [10]. Less than a year later, *PALB2* had already been recognized as an important gene for breast cancer susceptibility and, later, also for pancreatic cancer [11,12,13,14,15]. Only in 2009, PALB2 was identified as a linker protein for BRCA1 and BRCA2 [16,17,18].

PALB2 is a pivotal player of the DNA damage repair; especially by its participation on the Fanconi anemia (FA) pathway; biallelic *PALB2* mutations cause the N subtype of Fanconi anemia (FA-N). PALB2 also plays a critical role in homology-directed repair via the modulation of BRCA2 and RAD51 recruitment to double strand break (DSB) sites [10,17,19].

Here, we will address the most important aspects of PALB2 biology. From the role in DNA damage repair to the impact of gene mutations in cancer predisposition, passing through PALB2 molecular features and interaction partners depicting its role as a tumor suppressor gene.

## 2. PALB2 in Cancer Predisposition and Clinical Management

### 2.1. Cancer Predisposition

Shortly after its description as a BRCA2 protein interactor, *PALB2* was defined as a FA and BC susceptibility gene [19,20,21]. Rahman and colleagues (2007) reported that *PALB2* germline truncating variants occurred in 1.1% of patients from a subset of familial BC cases which tested negative for *BRCA1* and *BRCA2* alterations [21]. In the same study, *PALB2* was reported to have an incomplete penetrance pattern, typical of moderate cancer risk susceptibility genes, and was estimated that the relative risk for *PALB2* truncating variant carriers was 2.3-fold higher than non-carriers [21]. In later studies, *PALB2* truncating variants were reported to contribute to a 2–30-fold higher risk of BC incidence compared to non-carriers, depending on the mutation and population analyzed [15,22,23,24].

In Rahman and colleagues′ description, a *PALB2* mutation was reported in one family with a male BC case, suggesting the involvement of *PALB2* in male BC predisposition. Accordingly, germline *PALB2* truncating mutations in male BC cases were also reported in other studies [21,25,26,27]. Pritzlaff and colleagues (2017) estimated an increased risk of developing male BC in *PALB2* truncating mutation carriers (odds ratio, OR = 6.6; *p* = 0.01). For comparison, male BC risk associated to *BRCA2* and *CHEK2* pathogenic variants was reported to be 13.9 (OR) and 3.7, respectively [28].

*PALB2* germline pathogenic variants have been described with variable frequencies in BC patients depending on the studied population. For unselected BC cohorts, frequencies range between 0.86% in Asian and 0.93% in Polish patients [29,30]. Prevalence of *PALB2* pathogenic variants is higher in familial and/or hereditary BC cases, varying from 0.36% in a French cohort to 4.8% in Finnish patients; the high index observed in Finland is attributed to the c.1592delT founder mutation [31,32]. Differently from *BRCA1* and *BRCA2*, *PALB2* truncating mutations may not be associated with BC incidence in Jewish Ashkenazi population, heretofore no truncating variants have been identified in patients from this group [33,34]. Likewise, no evidence of *PALB2* association with BC was seen in Irish, Japanese or Dutch studies that evaluated patients at risk for hereditary breast and/or ovarian cancer [35,36,37].

Taking into account the well-established association of *PALB2* with BC and its close relationship with *BRCA1* and *BRCA2*, it is legitimate to inquire whether mutations in this gene would enhance the risk for ovarian cancer (OC). *PALB2* germline truncating variants were observed in 0.2–0.6% of women diagnosed with ovarian, fallopian tube and peritoneal carcinoma [38,39,40,41]. However, Southey and colleagues (2016) found no association between *PALB2* germline variants and OC in a multicenter case-control study [15]. In view of this cloudy scenario, there is no sufficient evidence for the association of *PALB2* germline truncating variants with OC risk.

Several reports in the literature indicate the association of *PALB2* germline truncating variants with pancreatic cancer (PC) [25,42,43,44,45,46]. Frequencies of ~4% have been described in familial PC patients in European, Australian and Japanese cohorts [43,44,47,48]. Very much alike to what was observed for BC, *PALB2* PC association seems to be dependent on the population studied, e.g., no pathogenic variant was found in Dutch or Italian PC patients [37,49]. Potentially pathogenic *PALB2* variants were also found in patients with other cancer types, like stomach/gastric, prostate and colorectal—although there is no association study linking *PALB2* to these tumors [25,50,51,52,53,54].

*PALB2* cancer association studies were mainly focused on truncating mutations, but the presence of missense variants of unknown significance (VUS) has also been reported in patients [31,34,36,55,56]. Recently, Foo and colleagues (2017) identified the missense variant L35P in a family with a strong history of BC and described it as a pathogenic variant [57].

### 2.2. Clinical Management

*PALB2* has emerged as a relevant cancer susceptibility gene and assessment of *PALB2* variants is informative in genetic counseling of patients at high risk of developing cancer. Recent findings point out that identification of *PALB2* variants in patients may also benefit clinical management in prognosis and treatment.

*PALB2* germline truncating variants associated with BC display an aggressive tumor phenotype, showing higher tumor grade and also higher levels of the proliferation marker protein Ki-67 [58]. It is worth noting that nearly 40% of the *PALB2* truncations associated with BC cases display a triple-negative phenotype, regardless of a specific mutation [59]. It is also interesting that *PALB2* was observed more frequently mutated in metastatic BC when compared to early cases [60]. Therefore, *PALB2* truncating mutations may predict a worst prognosis scenario in BC patients.

There is evidence suggesting that *PALB2* mutations could also be predictors of therapy response. Villarroel and colleagues (2011) described a pancreatic cancer patient that had a long-lasting response to mitomycin C (MMC, a crosslinking agent) treatment and whose tumor was deficient for *PALB2* [61]. Sensitivity to MMC due to *PALB2* mutations had previously been reported in lymphoma cell lines [62].

The presence of germline and somatic mutations in genes involved in homologous recombination (HR), including *PALB2*, was highly predictive of primary platinum (another group of crosslinking agents) sensitivity (*p* = 0.0002) and improved overall survival (*p* = 0.0006) in patients with ovarian, fallopian tube and peritoneal carcinoma. Median overall survival was of 66 months for germline HR-associated gene variant carriers, 59 months for somatic cases, and 41 months for cases without genetic alteration [40]. In Spugnesi and colleagues’ report (2016), germline variants in DNA repair genes, including *PALB2*, were found to be associated with the group of triple negative BC patients who responded to neoadjuvant therapy using anthracyclines and taxanes [63]. *PALB2* pathogenic variants were reported to be sensitive to PARP inhibitor treatment, similar to *BRCA1* and *BRCA2*. Goodall and colleagues (2017) evaluated circulating free DNA from prostate cancer patients treated with the PARP inhibitor olaparib in a phase II clinical trial [64]. Patients that carried somatic mutations in HR genes (*ATM*, *BRCA2* and *PALB2*) were responders of olaparib treatment. Interestingly, in progressive disease, following drug response, subclonal aberrations reverting germline and somatic mutations in *BRCA2* and *PALB2* were observed, suggesting a possible mechanism of resistance [64].

## 3. Gene and Protein Structures

**Figure 1:**
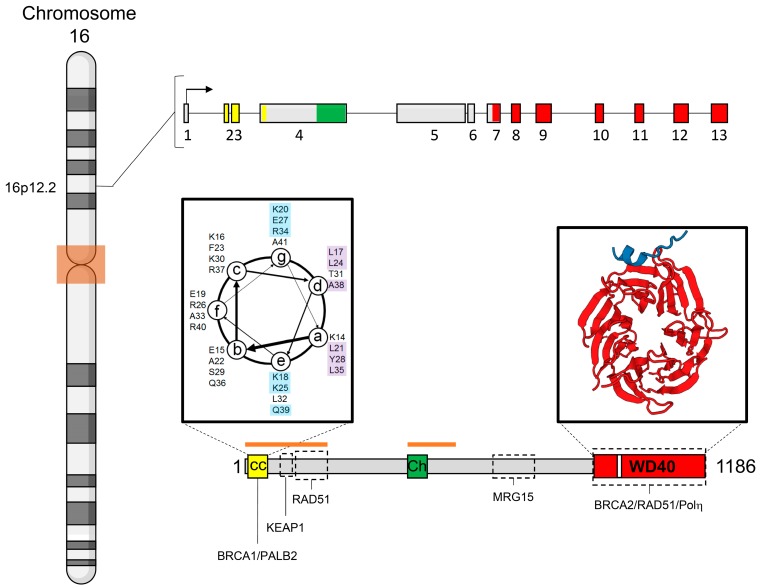
*PALB2* gene and its product. *PALB2* locus. Graphic representation of 16p12.2 chromosome; darker lines and ochre-colored box represent chromosome cytobands and centromere, respectively. Schematic diagram of *PALB2* gene organization. *PALB2* is composed of 13 exons; exons 2, 3 and part of the fourth encode the coiled-coil motif (depicted in yellow); the 3′ end of exon 4 codes for the Chromatin-associated motif (ChAM; in green), while the WD40 domain is encoded from the 3′ end of exon 7 to exon 13 (in red). PALB2 protein structure. PALB2 is an 1186 amino acid residue protein that includes a conserved coiled-coil motif at its amino-terminal region (CC; yellow box). The left inset represents the CC organization as a left-handed alpha helix, starting with the K14 residue and ending at the A41 residue, after four turns. Amino acids with acid lateral chains are depicted in blue and the ones with hydrophobic chains are in purple. PALB2 also includes two DNA binding regions (orange bars), a conserved motif responsible for binding to nucleosome structures (ChAM, in green) in its central portion and a WD40 domain (in red) at the carboxy-terminal region. The white stripe in the WD40 domain indicates its cryptic nuclear export signal. The right inset represents the WD40 domain (from NGL viewer of Research Collaboratory for Structural Bioinformatics (RCSB) Protein data bank—Peptide ID: 3EU7), depicting the β-propeller secondary structure, its seven blades (in red) and the interaction with the of BRCA2 amino terminal region (from amino acid residue 21 to 39; in blue) [71,72,73]. It is noteworthy that BRCA2 interacts with the pocket between the fourth and fifth blades of WD40 domain. Dashed boxes determine PALB2 interaction regions with different partners. Orange lines indicate the DNA-binding regions of *PALB2*.

*PALB2* (OMIM accession # 610355) is encoded by a 38 Mb gene composed of 13 exons located on the short arm of the chromosome 16 (16p12.2) (Figure 1). Little is known about the organization of *PALB2* promoter elements or gene expression regulation. However, it was recently proposed that the promoter region of *PALB2* presents a putative G-quadruplex structure, similar to *c-myc* and *HIF-1α* [65]. Such arrangement is related to downregulation of gene expression [66,67,68]. *PALB2* predicted promoter region was mostly observed hypermethylated in tumors from an Australian cohort of hereditary breast cancer cases [69]. Interestingly, in Potapova’s (2008) study, the methylation frequency observed in breast and ovarian cancers (hereditary and sporadic) was described as similar to the one observed in *BRCA1* promoter region [70]. The data support the idea of a transcriptional downregulation of *PALB2* in cancer cells, suggesting that *PALB2* silencing could be an important mechanism related to tumorigenesis.

*PALB2* encodes an 1186-amino acid protein with a conserved coiled-coil motif in its amino-terminal region (Figure 1). A coiled-coil is usually composed of 2–5 left-handed α-helices in a supercoiled structure [74,75]. According to COILS, a coiled-coil predictor tool [76], PALB2 presents a four-helix coiled-coil respecting the peptide velcro hypothesis, with prevalence of hydrophobic amino acid residues at “a” and “d” positions and of charged ones at “e” and “g” positions (Figure 1) [77]. PALB2 coiled-coil is responsible for mediating its homodimerization and its heterodimerization with BRCA1, which will be discussed further in this review [16,17,18,78,79]. RAD51, the recombinase responsible for the error-free repair by HR also interacts with PALB2 through its amino-terminal region (residue 101 to 184), which leads to the enhancement of RAD51 activity [80].

It was demonstrated that PALB2 presents two DNA-binding regions (Figure 1), which are responsible for stimulating DDR functions in FA and HR pathways through association with D-loop and single strand DNA (ssDNA) structures [80,81]. PALB2 also displays an evolutionary conserved chromatin-association motif (ChAM) in its central region (Figure 1) which is responsible for PALB2 association with chromatin through the nucleosome core histones H3 and H2B but not with secondary DNA structures [82]. The reconstitution of *PALB2* deficient cells derived from a FA-N patient with PALB2 ΔChAM did not rescue the sensitility phenotype toward MMC treatment, suggesting that PALB2 chromatin association via ChAM facilitates PALB2 function in cellular resistance to DNA damage [82].

In its carboxyl-terminal region, PALB2 presents a WD40 domain (Figure 1), which is canonically characterized as a β-propeller composed of seven repeats of 40 to 60 amino acid residues with the signature WD dipeptide at the end of every repeat [83,84]. BRCA2 interacts with PALB2 through the WD40 domain, more specifically via a pocket formed between the fourth and fifth repeats (depicted in Figure 1 WD40 domain) [73]. PALB2 variant A1025R, positioned at the bottom of the pocket, completely abrogates the interaction with BRCA2 [84]. Furthermore, PALB2 interaction with RAD51 is also mediated by PALB2 WD40 domain [80].

Pauty and colleagues (2017) demonstrated that the WD40 domain hides a nuclear export signal (NES) enclosed in the β-propeller structures (Figure 1). It was shown that the truncating mutation Y1038X was responsible for PALB2 translocation from the nucleus to the cytoplasm due to the exposure of the putative NES (residues 928–945) hidden in the WD40 domain [85]. These authors proposed that pathogenic mutations located in this region could expose the signal and then translocate PALB2 to the cytoplasm, preventing it from playing its role on DNA integrity maintenance and consequently prompting cells to tumorigenesis [85].

PALB2 acts as a hub in the DDR and its protein domains play an important role in mediating interactions, consequently transducing the DNA repair signal. Therefore, the integrity of these protein domains is essential for the maintenance of PALB2 tumor suppression function.

## 4. PALB2 in Fanconi Anemia and Homologous Recombination

PALB2 displays its molecular functions in the DDR through two intimately connected pathways, the FA and the HR repair.

### 4.1. Fanconi Anemia

FA is a rare autosomal or X-related recessive genetic condition characterized by defects in the DNA repair FA pathway. Individuals with a pathogenic variant in homozygosis on *FANC* genes, such as *PALB2* (Fanconi Anemia Group N protein; *FANCN*), develop the FA syndrome [20]. A hallmark of FA patients/cells is genomic instability and hypersensitivity to crosslinking agents. Affected carriers commonly develop bone marrow failure, have specific congenital abnormalities and have an increased risk of developing cancer [86].

The products of the FA pathway genes can be grouped into separate clusters based on their functions in interstrand crosslink (ICL) repair. According to Wang’s classification [87], Group 1 includes the core complex, and consists of 14 proteins: FANCA, FANCB, FANCC, FANCE, FANCF, FANCG, FANCL, FANCM, and FANCT; along with FA-Associated Proteins (FAAP): FAAP100, FAAP20, FAAP24, MHF1 and MHF2 [88].

**Figure 2:**
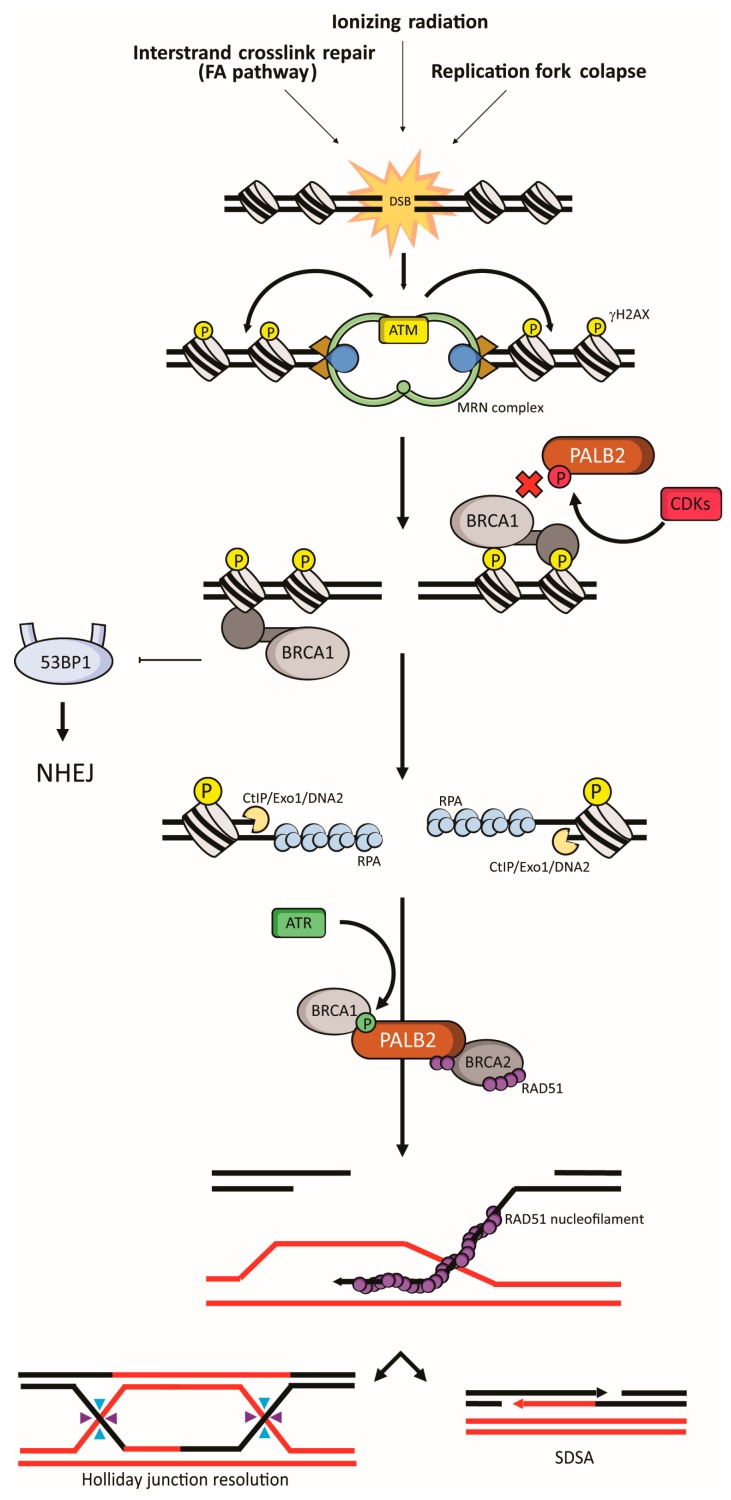
The role of PALB2 in the DNA damage repair. Schematic representation of double strand break (DSB) induction and resolution throughout the HR repair pathway. Double strand breaks can result from different sources (as a by-product of the FA pathway, ionizing radiation treatment or replication fork collapse in response to replicative stress). After the DSB is induced, the MRN (MRE11-RAD50-NBS1) sensor complex recognizes and binds this structure. ATM is then recruited and activated, leading to the phosphorylation of H2AX (γH2AX), amplifying the initial signaling. The signal transduction leads to BRCA1 recruitment to the DNA damaged site, driving the HR repair by inhibition of 53BP1 and consequently the Non Homologous End-joining (NHEJ) pathway. At this point, PALB2 is found phosphorylated (pS64; in red) by Cyclin-dependent Kinases (CDKs), which prevents its interaction with BRCA1. Then, the 5′ DNA end resection is performed by C-terminal binding protein (CtBP) Interacting protein (CtIP), Exonuclease 1 (Exo1) and DNA2 exonuclease activities. The replication protein A (RPA) caps the exposed ssDNA, culminating in the activation of ATR and PALB2 (pS59; in green), which favors PALB2-BRCA1 interaction (in contrast with pS64). Thus, BRCA1 recruits PALB2, BRCA2 and RAD51, promoting exchange of RPA by RAD51, nucleofilament formation and strand invasion. Later, the repaired DNA is resolved in a Holliday junction-mediated (light blue and purple arrowheads represent the crossover and non-crossover resolution types, respectively) or by synthesis dependent strand annealing-dependent (SDSA) manner.

FANCM recognizes ICLs during DNA replication and functions as a recruiting platform for the core complex. Although its functions are not completely elucidated, the core complex multimerization is necessary for monoubiquitylation of the Group 2 proteins FANCD2-FANCI [89]. This monoubiquitylation is catalyzed by the core complex and the ubiquitin ligase FANCL and its E2 ubiquitin-conjugating enzyme UBE2T/FANCT [90,91]. Group 3 is composed of effectors downstream of FANCD2-FANCI [88]. After the loading of FA proteins on the chromatin and lesion recognition, ubiquitilated FANCD2-FANCI regulates nucleolytic incision at the intersect of replication forks to release the ICL from one of the two parental DNA strands [92]. FANCD2, in turn, recruits the Group 3 nucleases SLX4/FANCP, ERCC1-ERCC4 (also known as XPF or FANCQ) heterodimer, MUS81–EME1 and SLX1 [93,94]. Group 3 also includes HR proteins (BRCA2/FANCD1, BRIP1/FANCJ, PALB2/FANCN, RAD51C/FANCO, RAD51/FANCR, BRCA1/FANCS, and XRCC2/FANCU) and the translesion synthesis (TLS) factor REV7/FANCV. Once the lesion has been unhooked from one of the DNA strands, the missing nucleotides in this strand are incorporated by the low-fidelity TLS DNA polymerases (process known as “insertion”) and extending the nascent strand (“extension”). This step potentially introduces mutations in the restored strand [95]. This strand is then used as a template for repair by HR of the broken DNA duplex left (Figure 2).

### 4.2. Homologous Recombination

HR is triggered by DSB in replicating cells (S and G2 phases), as consequence of an original damage (e.g., by ionizing radiation, IR) or downstream of the FA response to an ICL insult. HR is orchestrated by the kinases ATM and ATR [96]. Both kinases are responsible for the phosphorylation of a large number of proteins related to different steps in this pathway [96]. ATM is activated by the MRN sensor complex (MRE11, RAD50 and NBS1) that recognizes DSB structures (Figure 2) [97]. The sensor protein RPA is responsible for recognizing ssDNA, and recruiting TopBP1 that drives ATR activation [98]. ATR recruitment can be the result of a primary damage (e.g., by replication stress) or a downstream intermediate in response to DSBs. During the DSB repair, ATM stimulates the process known as 5′ DNA end resection, which exposes ssDNA that triggers ATR (Figure 2). DNA end resection is initiated by MRN complex and later processed by C-terminal binding protein (CtBP) interacting protein (CtIP), Exonuclease 1 (Exo1) and DNA2 [99]. After the ssDNA capping by RPA, BRCA1 recruits BRCA2 and RAD51 through the coiled-coil-mediated interaction with PALB2. Therefore, the absence of PALB2 leads to impaired loading of BRCA2 and RAD51 to DSB sites [16,17,18]. This complex mediates the RPA replacement by RAD51 and consequently the RAD51-dependent D-loop formation, RAD51 nucleoprotein filament polymerization and strand invasion. It was also demonstrated that PALB2 is capable of binding ssDNA and D-loop structures, interacting directly with RAD51, placing PALB2 as a critical hub in FA/HR-mediated repair [79,80].

It is not clear how the elongation process for the late resolution of HR is conducted but it has been shown that TLS DNA polymerases (Polδ, Polη and Polκ) act in D-loop elongation [100,101,102]. PALB2 binds to Polη through the WD40 region (Figure 1). PALB2 and BRCA2 interact with Polη, and are required to sustain its recruitment at stalled replication forks, stimulating Polη-dependent DNA synthesis on D-loops, suggesting a role for PALB2 in later steps of HR [103]. The repair can then be resolved by a Holliday junction intermediate or by synthesis-dependent strand-annealing (SDSA) (Figure 2) [104,105].

## 5. Regulation of PALB2 Functions

Literature places PALB2 as an important player in different steps of HR repair [16,17,18,77,78,80,81,103]. It is reasonable to assume that PALB2 post-translational modifications operate key mechanisms in HR and DDR control. An important regulation of HR occurs via PALB2 phosphorylation. PALB2-BRCA1 interaction is modulated by ATM, ATR and CDKs during S/G2 phases, controlling the homology-directed repair.

After a DSB, PALB2 is initially phosphorylated by CDKs at serine 64 (S64), as well as CtIP. In fact, both phosphorylations are attributed to CDKs due to the consensus phosphorylation motif (S/T-P) observed in both targets [106]. While CtIP phosphorylation triggers DNA 5′ end resection, PALB2 S64 phosphorylation prevents BRCA1 interaction (Figure 2 depicts PALB2 S64 phosphorylation in red). The exposure of ssDNA leads to RPA recruitment and ATR activation, driving CDKs inhibition and phosphorylation of PALB2 at serine 59 (S59), which favors PALB2-BRCA1 interaction (Figure 2 depicts PALB2 S59 phosphorylation in green). Buisson and colleagues (2017) demonstrated that the PALB2 phosphomimetic mutant S59E/S64A co-localizes with BRCA1 in DNA damaged sites in the presence of ATR inhibitors [107].

PALB2 is also modulated by ATM phosphorylation in response to IR treatment at serine residues 157 (S157) and 376 (S376). The S157 phosphorylation, but not S376, occurs in a BRCA1-PALB2 interaction-dependent manner. Interestingly, in a gene conversion assay, phosphorylation of both serine sites seems unnecessary for DNA damage repair through HR [108]. However, the PALB2 triple mutant (S59A, S157A and S376A) presents impaired RAD51 recruitment to DNA damage sites [109]. It is noteworthy that the notion presented here, i.e., PALB2 is a substrate of different kinases, such as ATM, ATR and CDKs, illustrates its role as a hub in the DNA damage repair by HR.

**Figure 3:**
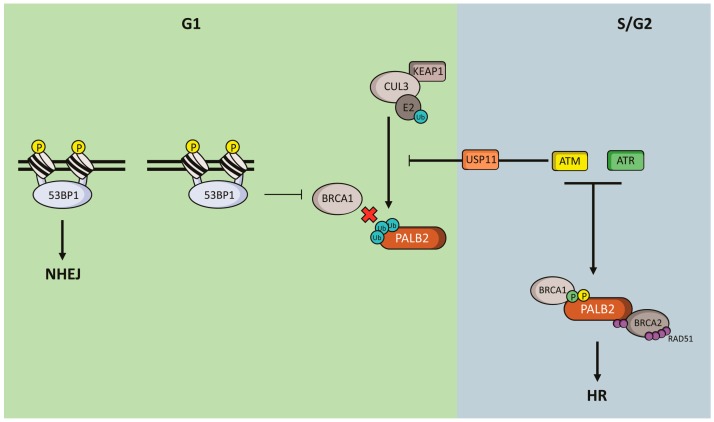
Cell cycle-mediated PALB2 regulation. Schematic representation of G1-dependent PALB2 ubiquitylation conducted by the CUL3 E3 ubiquitin ligase complex, mediated by KEAP1-PALB2 interaction. During the G1 phase (green background), 53BP1 inhibits BRCA1 recruitment to DSB sites, favoring NHEJ repair. Homologous recombination repair is also repressed through the inhibition of BRCA1-PALB2 interaction, a consequence of PALB2 ubiquitylation. In S/G2 (blue background), DNA damage activates the ATM-USP11 axis of DDR, which leads to the inhibition of the PALB2 ubiquitylation and consequently favors its association with BRCA1. Further, both ATM and ATR also phosphorylate PALB2, stimulating the DNA repair by HR through the BRCA1-PALB2-BRCA2-RAD51 complex.

An additional mechanism of HR modulation occurs via the cell cycle-regulated DNA end resection event, which is based on the recruitment balance between BRCA1 and 53BP1 [110]. In G1, HR is repressed by 53BP1 through the inhibition of BRCA1 recruitment to DNA damage sites. This idea is supported by the observation of BRCA1 recruitment to DNA damaged sites in cells lacking 53BP1 during the G1 phase. In a counterintuitive way, neither PALB2 nor BRCA2 are recruited to DSBs in the absence of 53BP1 [111]. These phenomena are mechanistically explained by the cell cycle-regulated ubiquitylation of PALB2. In G1 cells, KEAP1 (a previously characterized PALB2 partner, Figure 1) recruits CUL3, which catalyzes the monoubiquitylation of PALB2 N-terminal region on lysine residues K20, K25 and K30 (Figure 3). PALB2 ubiquitylation inhibits BRCA1 interaction, and consequently PALB2 and BRCA2 recruitment to DNA damaged sites, even in a 53BP1-deficient scenario [111,112]. In contrast, in S/G2 phases, irradiated cells activate the ATM-USP11 axis of DNA damage checkpoint that drives the deubiquitylation of PALB2, favoring the formation of the BRCA1-PALB2-BRCA2 complex and thus the repair by HR. These observations place the BRCA1-PALB2 complex formation as a central step of the HR repair inhibition in non-cycling cells, besides the DNA end resection step (Figure 3).

Among the mechanisms involving PALB2 modulation, MRG15 (coded by *MORF4L1* gene) rises as an important interactor and regulator of PALB2 functions during HR repair [81,113,114,115]. MRG15 was first described as a component of the histone acetyltransferase complex NuA4, which was recently associated with the recruitment of BRCA1 by the acetylation of histone H4 and H2A in an ATM-ACLY axis-dependent manner [116,117]. Before its characterization as a PALB2 interactor, MRG15 had already been associated with defects in proliferation, DNA repair and recruitment of DDR-related proteins in a mouse embryonic fibroblast model [118,119]. MRG15 and its homolog MRGX were identified as PALB2 interactors in tandem affinity purification followed by mass spectrometry (TAP-MS). Cells expressing a PALB2 dominant negative (Δ611–764) for the PALB2/MRG15 interaction, as well as cells lacking MRG15, presented a hyper-recombination phenotype [114]. Besides PALB2, MRG15 also interacts with BRCA2 and RAD51, being required for their recruitment to DSB sites and consequently to the HR repair [113]. MRG15 is also responsible for PALB2 loading onto active genes, preventing replicative stress in these regions [120]. Based on these functions in the HR repair, different groups tried to identify a possible link of *MORF4L1* with cancer susceptibility. Nevertheless, there is no strong evidence of a connection between *MORF4L1* and breast cancer susceptibility [121,122].

It was first proposed that PALB2 could regulate DNA damage repair through its coiled-coil-mediated self-interaction [78]. However, it became clear that the phenotype observed by Sy and colleagues (2009) was due to the abrogation of BRCA1-PALB2 interaction, later shown by the same group and others [16,17,18]. PALB2 homodimerization inhibits its association with BRCA1, as this interaction is mediated by the same motif. Moreover, BRCA1 coiled-coil domain can disrupt PALB2 self-interaction [77]. It was also shown that a truncated form of PALB2, missing its first forty amino acid residues, presented an enhanced DNA binding but had no effect on the RAD51-mediated D-loop formation. Additionally, the overexpression of the first 200 amino acids of PALB2 was enough for the inhibition of RAD51 DNA foci formation, while a Δcoiled-coil form of the protein had no effect on RAD51 recruitment to DSB sites [77]. The data suggest that this mechanism might modulate HR repair through the inhibition of BRCA1-PALB2 interaction. Further studies are necessary to understand the precise role of PALB2 homodimerization and its impact in modulating the DDR [77,107,108,109,111].

## 6. PALB2 and RAD52

The DNA damage response arose throughout the evolutionary process as a major feature in genome integrity maintenance and consequently tumorigenesis suppression in more recent eukaryotes [123]. Despite the conservation of the DDR pathway in higher eukaryotes, important DSB repair-associated proteins such as BARD1 (BRCA1 associated RING domain 1) and PIAS1 (protein inhibitor of activated STAT1) emerged only before the split of plants. PALB2 appeared long after, immediately before the emergence of vertebrates, suggesting a distinct role for this protein on DSB repair [124]. In *Saccharomyces cerevisiae*, ScRad52 is a central protein in the regulation of DSB repair by HR [125,126]. During HR, ScRad52 coordinates two important steps, including the assembly of ScRad51 filament on RPA-coated ssDNA and the annealing of complementary DNA strands [127,128,129]. Corroborating the biological relevance of ScRad52, null mutants display defective DNA repair and severe sensitivity to DNA damage-inducing agents [130,131]. Remarkably, mammalian RAD52 function is not as central as its fungal counterpart, with knockout mice showing nearly no phenotype in DNA repair and DNA damage sensitivity [132]. Differently from the yeast ortholog, RAD52 does not regulate RAD51 binding to ssDNA, and is therefore usually reported as a functional ortholog of ScRad59 [133,134,135,136]. In vertebrates, RAD51 assembly to ssDNA is taken over by different RAD51 paralogs or BRCA2 [137]. Indeed, according to Jensen and colleagues (2010), BRCA2 acts as the functional ortholog of ScRad52, since it stimulates RAD51 recombinase activity [137,138]. However, differently from ScRad52, BRCA2 is not capable of annealing RPA-coated ssDNA [137,138,139]. It is reasonable to speculate that during evolutionary processes ScRad52 might have been substituted by other factors with the ability to complement RAD51 and BRCA2 functions. In vitro data show that PALB2, even in the absence of BRCA2, can potentiate RAD51 strand exchange [80]. Furthermore, PALB2 also stimulate RAD51-mediated D-loop formation in an ATP-dependent and BRCA2 independent manner [79]. Additionally, PALB2 also plays an important role in replication fork recovery. During the replication stress response, ATR-dependent phosphorylation of RPA recruits PALB2 and BRCA2 to stalled replication forks in order to promote fork recovery. In yeast, a similar role is played by MRX-Rad52-rfc1 [140,141]. It is noteworthy that RAD52 inactivation is synthetically lethal toward PALB2 or BRCA2 deficiency in human cells, reinforcing the idea of RAD52 acting in a distinct pathway and supporting the hypothesis of PALB2 as a functional ortholog of ScRad52 [142,143].

## 7. Concluding Remarks

Here, we presented a current view of the most important features and functions of PALB2 in genome integrity maintenance and tumorigenesis suppression. *PALB2* emerged in the literature as a moderate-risk BC susceptibility gene; however, recent studies already report *PALB2* as a high-risk gene [23,24].

The contribution of *PALB2* pathogenic variants is already recognized in clinical oncology; a large number of truncating variants have been reported to be associated with different forms of cancer, but a significant number of missense variants remain unclassified. It is known that PALB2 cancer-related missense variants L393W, T1030I and L1143P in the WD40 domain could interfere with PALB2 association with RAD51 and BRCA2, prompting carriers to be defective in DNA damage repair by HR, leading to an increased sensitivity to IR-treatment [144]. Similarly, PALB2 L35P pathogenic variant abrogates PALB2 association with BRCA1 [57]. Therefore, evaluating *PALB2* VUS impact in cancer predisposition is central for risk assessment. Validated functional assays that interrogate individual alleles for specific molecular functions constitute robust tools for clinical annotation [61]. Determining the behavior of a PALB2 variant using functional approaches such as protein interaction or proficiency in HR repair comprise an alternative to unravel its clinical significance. However, genetic linkage evaluation is still recommended for the validation of risk association.

As PALB2 links BRCA1 and BRCA2 and mutations in this gene is associated to susceptibility to breast cancer, it became a natural question whether *PALB2* mutations could also lead to OC. This association is still inconsistent and it remains to be better evaluated [15]. Overall, significantly less OC cases are seen in *PALB2* families when compared with *BRCA1* and *BRCA2* families [145]; therefore, it seems that *PALB2* may contribute in a different way than *BRCA1* and *BRCA2* to cancer predisposition, possibly as a result of PALB2 distinct roles in DDR.

Since the first observation of PALB2 interacting with BRCA1 and BRCA2, PALB2 has drawn attention to its functions in the DDR, mainly through the repair of DSBs [16,17,18]. The accumulated literature places PALB2 in a central position of the DNA damage repair by the FA/HR pathway, as a switch panel that must be finely regulated in order to drive the repair of DSB through HR [10,16,17,18,107]. This control is mediated by regulation of PALB2 phosphorylation, ubiquitilation and homodimerization; evidence supports that these modulations are essential for genomic integrity [77,78,107,109,111,112]. The inhibition of these signalizations could be a mechanism for sensitizing cells to DNA damaging agents, placing PALB2 as a putative target for cancer treatment.

PALB2 modulation could also be involved in BC progression. Next generation sequencing of both primary and metastatic tumors from an estrogen-receptor-alpha-positive metastatic lobular BC case revealed that, from the 32 somatic non-synonymous coding mutations present in the metastatic sample, five were prevalent in the DNA of the primary tumor, including a somatic *PALB2* mutation [146]. It is reasonable to infer that these mutations may have contributed to disease progression, indicating a relevant role of *PALB2* in preventing tumorigenesis.

The knowledge on molecular functions of PALB2 tumor suppressor is still emerging and, eventually, points to PALB2 as a therapeutic target. However, it is still not clear whether PALB2 affected carriers could benefit from a specific treatment, such as the PARP inhibitor therapy [61,80].

We also discussed here the role of PALB2 as a functional ortholog of ScRad52, being essential for the proper function of RAD51. PALB2 deficient cells present a switch from the error-free (HR) to the mutagenic resection-dependent repair (single strand annealing and microhomology-directed repair) which might explain the genomic instability phenotype acquired in PALB2 mutated tumors [57,147]. Thus, it is reasonable to hypothesize that PALB2-deficient cells would be more sensitive to RAD52 inhibitors, as seen for BRCA1 or BRCA2 [148].

Despite all the evidence for the biological functions of PALB2, further studies are needed to help unravel the underlying principles of PALB2 in cellular response to DNA damaging insults and the consequences for cellular fate.

## Abbreviations

ATM	Ataxia Telangiectasia Mutated
ATR	ATM- and Rad3-Related
BC	Breast cancer
BRCA1	Breast Cancer 1
BRCA2	Breast Cancer 2
CDK	Cyclin-Dependent Kinase
ChAM	Chromatin-associated motif
CtBP	C-terminal binding protein
CtIP	CtBP-Interacting protein
DDR	DNA damage response
DNA	Deoxyribonucleic Acid
DSB	Double strand break
FA	Fanconi anemia
FANCN	Fanconi Anemia Group N protein
HR	Homologous recombination
ICL	Interstrand crosslink
MMC	Mitomycin C
NES	Nuclear export sequence
OC	Ovarian cancer
OR	Odds ratio
PALB2	Partner and Localizer of BRCA2
PC	Pancreatic cancer
RCSB	Research Collaboratory for Structural Bioinformatics
RPA	Replication Protein A
SDSA	Synthesis dependent strand annealing
ssDNA	Single strand DNA
TAP-MS	Tandem affinity purification followed by mass spectrometry
TLS	Translesion synthesis
VUS	Variant of uncertain significance
